# Hydroxyurea alters circulating monocyte subsets and dampens its inflammatory potential in sickle cell anemia patients

**DOI:** 10.1038/s41598-019-51339-x

**Published:** 2019-10-15

**Authors:** Caroline C. Guarda, Paulo S. M. Silveira-Mattos, Sètondji C. M. A. Yahouédéhou, Rayra P. Santiago, Milena M. Aleluia, Camylla V. B. Figueiredo, Luciana M. Fiuza, Suellen P. Carvalho, Rodrigo M. Oliveira, Valma M. L. Nascimento, Nívea F. Luz, Valéria M. Borges, Bruno B. Andrade, Marilda S. Gonçalves

**Affiliations:** 1 Laboratório de Investigação em Genética e Hematologia Translacional, Instituto Gonçalo Moniz, FIOCRUZ-BA, Salvador, Bahia Brazil; 2 Faculdade de Medicina da Bahia, Universidade Federal da Bahia, Salvador, Brazil; 3 Laboratório de Inflamação e Biomarcadores, Instituto Gonçalo Moniz, FIOCRUZ-BA, Salvador, Bahia Brazil; 4 Multinational Organization Network Sponsoring Translational and Epidemiological Research (MONSTER) Initiative, Salvador, Bahia Brazil; 5 Curso de Medicina, Faculdade de Tecnologia e Ciências, Salvador, Bahia Brazil; 6 Fundação de Hematologia e Hemoterapia do Estado da Bahia (HEMOBA) Salvador, Bahia, Brazil; 7 Universidade Salvador (UNIFACS), Laureate Universities, Salvador, Bahia Brazil; 8 Escola Bahiana de Medicina e Saúde Pública, Salvador, Bahia Brazil

**Keywords:** Biomarkers, Inflammation

## Abstract

Sickle cell anemia (SCA) is a hemolytic disease in which vaso-occlusion is an important pathophysiological mechanism. The treatment is based on hydroxyurea (HU), which decreases leukocyte counts and increases fetal hemoglobin synthesis. Different cell types are thought to contribute to vaso-occlusion. Nevertheless, the role of monocytes subsets remains unclear. We investigated frequencies of monocytes subsets in blood and their response to HU therapy, testing their ability to express pro-inflammatory molecules and tissue factor (TF). We identified major changes in monocyte subsets, with classical monocytes (CD14^++^CD16^−^) appearing highly frequent in who were not taking HU, whereas those with patrolling phenotype (CD14^dim^CD16^+^) were enriched in individuals undergoing therapy. Additionally, HU decreased the production of TNF-α, IL1-β, IL-6, IL-8 as well as TF by the LPS-activated monocytes. Likewise, frequency of TF-expressing monocytes is increased in patients with previous vaso-occlusion. Moreover, activated monocytes expressing TF produced several pro-inflammatory cytokines simultaneously. Such polyfunctional capacity was dramatically dampened by HU therapy. The frequency of classical monocytes subset was positively correlated with percentage cytokine producing cells upon LPS stimulation. These findings suggest that classical monocytes are the subset responsible for multiple pro-inflammatory cytokine production and possibly drive inflammation and vaso-occlusion in SCA which is damped by HU.

## Introduction

Sickle cell anemia (SCA) is a genetic disease associated with important alterations of morphology and function of red blood cells (RBC) which cause a wide range of clinical manifestations linked to vascular injury and coagulation abnormalities^1^. The SCA is characterized by homozygosity of the hemoglobin S (HbS), and patients with this disease exhibit the most severe clinical forms^1^. Of note, polymerization of HbS triggers biochemical and morphological changes in sickle erythrocytes, which interact with other erythrocytes, as well as with reticulocytes, leukocytes, platelets and endothelial cells leading to vaso-occlusive events (VOE)^1,2^, which is the main pathophysiological mechanism underlying SCA. VOE is thought to be caused at least by three components: (i) activation of endothelial cells and leukocytes due to adherence of sickle erythrocytes; (ii) nitric oxide (NO) consumption by arginase and free hemoglobin as result of intravascular hemolysis; (iii) activation of coagulation cascades due to activation of endothelium and leukocytes, which drive blood flow obstruction and eventually VOE^1,3^. Understanding the mechanisms driving susceptibility to VOE is critical to develop optimization of clinical management and development of new therapeutic approaches of SCA patients.

Monocytes play an important role in innate immune responses. These cells originate from a myeloid progenitor from bone marrow, circulate in peripheral blood for approximately 2–3 days until they undergo apoptosis or migrate to the tissues where they become macrophages and maintain the innate immune surveillance^4,5^. They represent a very versatile leukocyte population which is responsible for a wide range of activities involved in immune defense against pathogens, maintenance of immune tolerance as well as of homeostasis^6^. Human monocyte subsets are characterized based on dichotomous expression of the surface markers CD14 and CD16 in classical (CD14^++^CD16^−^), intermediate (CD14^+^CD16^+^) and non-classical or patrolling (CD14^dim^CD16^+^) monocytes^7^. Such categorization is not stable and it has been shown that monocytes can turn from one subset to another depending on the microenvironment^8^. The diverse monocyte subsets exhibit distinct functions that can range from highly pro-inflammatory to immunosuppressant activities^9^. The involvement of monocytes subsets in the pathogenesis of several pathological scenarios has been evaluated, ranging from infectious diseases such as HIV infection^10^ and tuberculosis^11^ to inflammatory diseases such as atherosclerosis^12^ and myocardial infarction^13^.

In SCA, activated monocytes were shown to be associated with vascular dysfunction through different mechanisms. During vaso-occlusive crisis, monocytes activate endothelium by inducing nuclear factor-kappa B (NF-κB) translocation^14^. In addition, the direct contact with endothelial cells triggers upregulation of genes encoding adhesion molecules and cytokines^15^, aside from production of lipid mediators, adhesion molecules, and coagulation factors^2,16^, which may contribute to VOE. Importantly, increased levels of pro-inflammatory cytokines in SCA seem to be a critical factor contributing to onset of VOE. Elevated serum levels of TNF, IL-1β, IL-6 and IL-8 in SCA patients are correlated with endothelial cell activation, and increased cell expression of vascular cell adhesion molecule-1 (VCAM-1) and intercellular adhesion molecule-1 (ICAM-1), as well as of soluble forms of these molecules^17–19^.

Moreover, monocytes are a main source of tissue factor (TF)^20^, a critical molecule involved in activation of the extrinsic coagulation cascade leading to thrombin generation^20,21^. In a recent study, we have demonstrated that TF-expressing monocytes are in the epicenter of chronic inflammation and persistent activation of coagulation in patients living with HIV^10^. These cells produce multiple pro-inflammatory cytokines and are related to increased cardiovascular risk in HIV infection^10^. In SCA, expansion of monocytes producing TF has been reported during VOE^22^. The exact mechanisms by which TF-expressing monocytes may drive VOE and/or cardiovascular complications of SCA patients are not completely described.

Pharmacological treatment of SCA patients with severe clinical profile is based on hydroxyurea (HU) therapy, which has been associated to beneficial effects on the microvasculature and decreased occurrence of VOE and other clinical complications^23^. This drug exhibits cytostatic properties through the inhibition of ribonucleotide reductase, which stops cell division. Moreover, HU decreases neutrophil, monocyte and reticulocyte counts in peripheral blood, as well as the expression of adhesion molecules and cytokines; while increasing synthesis of fetal hemoglobin (HbF)^24^. Considering the intricate mechanisms related to SCA pathogenesis, we aimed to investigate in detail the effect of HU therapy on circulating monocytes subsets and on their ability to express TF as well as pro-inflammatory cytokines upon activation in SCA patients. Furthermore, we tested association between monocyte activation phenotypes and occurrence of VOE.

Our findings indicate that HU therapy induces substantial changes in frequency of monocyte subsets as well as in their capacity to promote inflammation and coagulation, which was associated to occurrence of VOE in SCA. Collectively, our data suggest that HU treatment modulate the inflammatory response driven by the monocytes.

## Results

### Impact of hydroxyurea therapy on laboratory parameters and clinical manifestations

The groups of participants were similar with regard to age and gender (Table S1). HU therapy was associated with improvement of most of biochemical and hematological parameters, including increases in hemoglobin levels and values of hematocrit, as well as reduction of LDH and AST concentrations. In addition, we observed a 2-fold increase in HbF levels and decrease of HbS levels (Table S1). HU use was also associated with decreased number of VOE, but no other change in clinical manifestations was noted in this specific study population (Table S2). The two patients underoing HU therapy experienced one episode of VOE six months prior to blood drawn and referred HU use for the last 6 years.

### Characterization of monocytes subsets of SCA patients under hydroxyurea therapy

**Figure 1:**
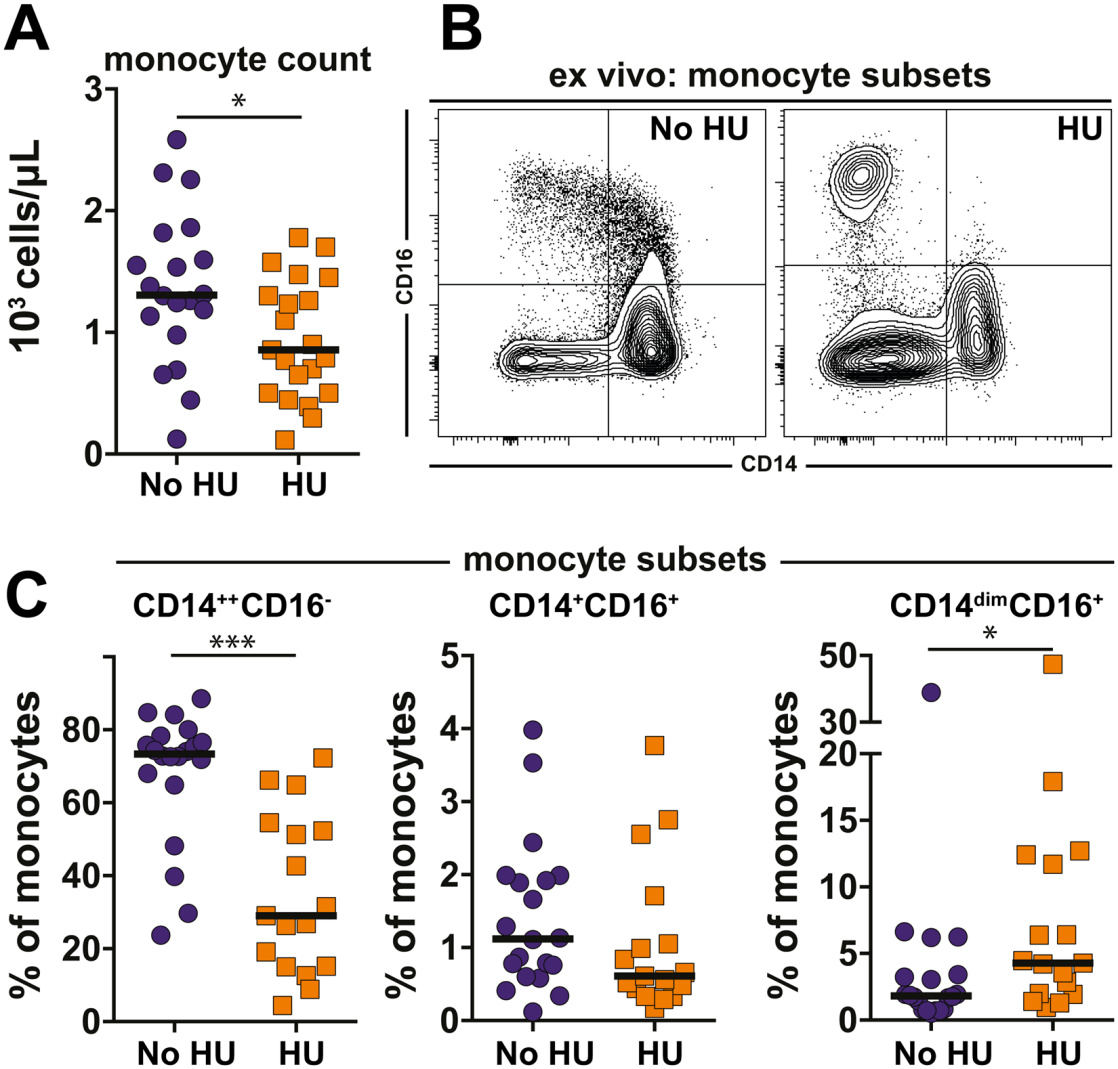
Hydroxyurea therapy induces major changes in peripheral blood monocyte subsets in patients with sickle cell anemia. (**A**) Total monocyte counts in blood examined by clinical cell counter in peripheral blood were compared between sickle cell anemia patients undertaking (n = 17) or not (n = 20) hydroxyurea using the Mann-Whitney *U* test. *p < 0.05. (**B**) Representative FACS plot of monocyte subsets examined by flow cytometry in PBMC. Overall gating strategy is shown in Supplementary Fig. 1. (**C**) Frequencies of indicated monocyte subsets PBMC between the study groups were compared using the Mann-Whitney *U* test. (HU group n = 17 and no HU group n = 20). *p < 0.05, ***p < 0.0001.

Monocyte counts were decreased in SCA patients undergoing HU therapy (Fig. 1A). We next performed multicolor flow cytometry assays to better define the effects of HU treatment on monocyte subsets. The experiments revealed that HU decreased frequency of CD14^++^CD16^−^ monocytes, while CD14^dim^CD16^+^ were increased compared with that of patients not undergoing HU therapy (Fig. 1B,C). No statistical significance was found in frequency of monocytes subsets expressing both CD14^+^ CD16^+^ between individuals undertaking or not HU. Altogether these results suggest that HU induces substantial changes in monocytes subtypes in peripheral blood.

### Modulation of cytokine production by monocytes driven by hydroxyurea

**Figure 2:**
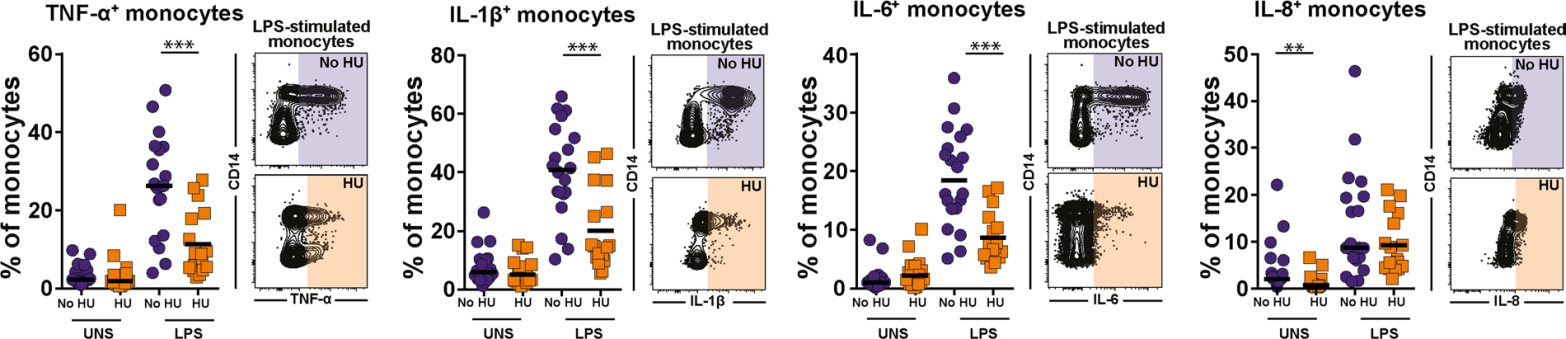
Hydroxyurea therapy negatively impacts production of pro-inflammatory cytokines of monocytes in response to LPS. PBMC from sickle cell anemia patients were incubated with 100 ng/mL LPS *in vitro* and intracellular cytokine staining assay was performed to test whether hydroxyurea treatment *in vivo* induces changes in the capacity of monocytes to respond to LPS by producing TNF-α, IL-1β, IL-6 and IL-8. Data represent frequency of monocytes. HU group n = 17 and no HU group n = 20. At each experimental condition, the study groups were compared using the Mann-Whitney *U* test. *p < 0.05, ***p < 0.0001.

We tested the effect of HU on cytokine production by monocytes. In unstimulated conditions, frequencies of monocytes expressing TNF-α, IL-1β or IL-6 were similar between the groups of patients taking or not HU (Fig. 2). Nevertheless, monocytes producing IL-8 were significantly expanded in patients not undergoing HU therapy (Fig. 2). Upon LPS challenge *in vitro*, monocytes were able to increase the production of TNF-α, IL-1β, IL-6 and IL-8 independent of the clinical group (Fig. 2). Importantly, HU use was associated with decreased capacity to produce TNF-α, IL-1β or IL-6 relative to that in patients who were not under HU therapy (Fig. 2). Production of IL-8 was not affected by HU treatment. These effects of HU were not linked to differences in cell death before and after LPS stimulation (data not shown).

### Effect of hydroxyurea treatment in tissue factor expression and vaso-occlusion events

**Figure 3:**
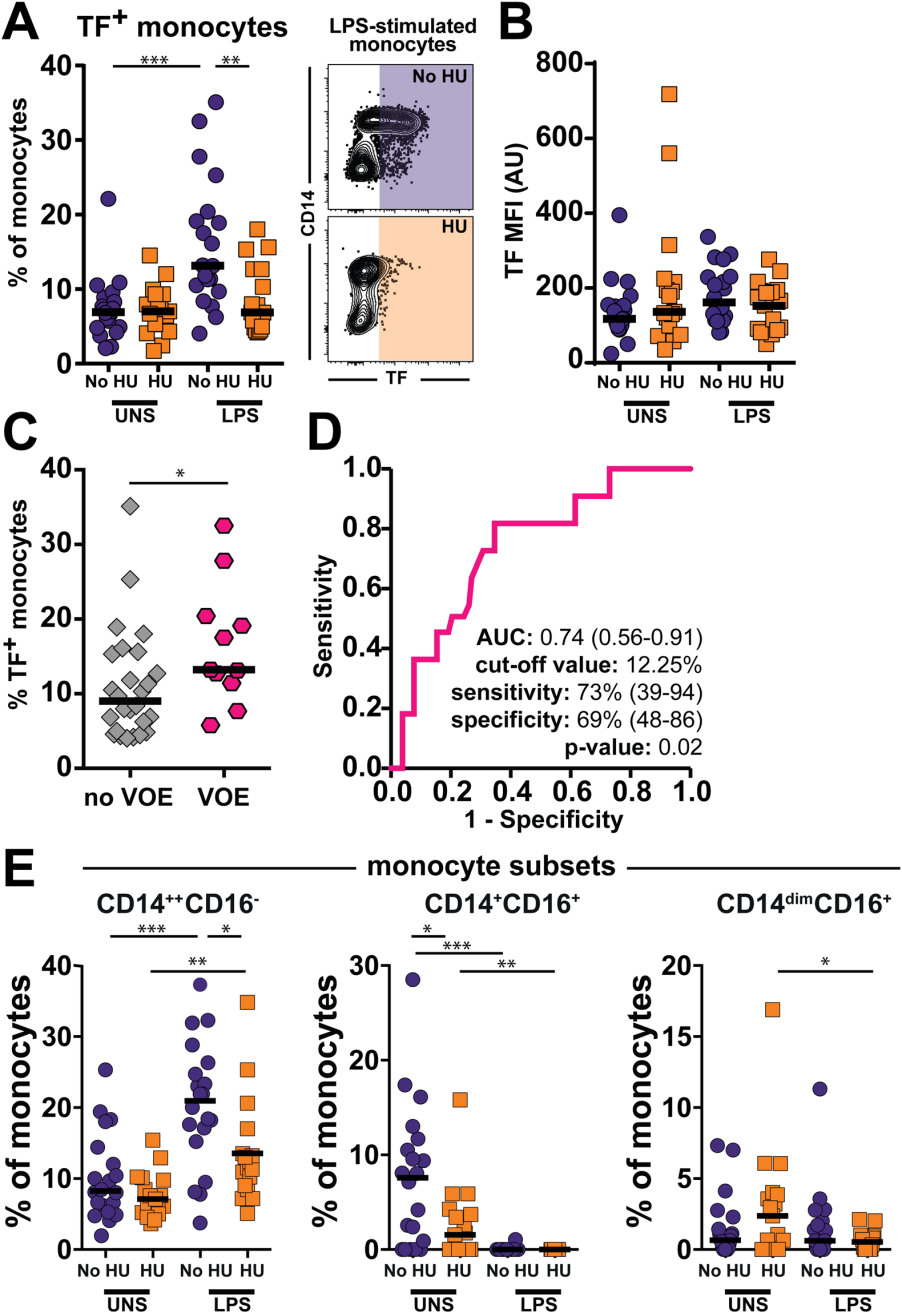
Sickle cell anemia-associated tissue factor production by monocytes in response to LPS is diminished by hydroxyurea treatment *in vivo*. (**A**) PBMC from sickle cell anemia patients were incubated with 100 ng/mL LPS *in vitro* and intracellular cytokine staining assay was performed to test whether hydroxyurea treatment *in vivo* induces changes in the capacity of monocytes to respond to LPS by producing tissue factor (TF). Data represent frequency of monocytes. HU group n = 17 and no HU group n = 20 At each experimental condition, the study groups were compared using the Mann-Whitney *U* test. **p < 0.01, ***p < 0.0001. (**B**) Mean Fluorescence Intensity (MFI) of TF expression by monocytes at indicated experimental conditions is shown. No statistically significant differences were observed. HU group n = 17 and no HU group n = 20. (**C**) Frequency of TF-expressing monocytes upon LPS stimulation was compared between SCA patients presenting or not previous occurrence of vaso-occlusive events (VOE). VOE group n = 11 and no VOE n = 26. The study groups were compared using the Mann-Whitney *U* test. *p < 0.05. (**D**) Receiver Operator Characteristics (ROC) curve analyses was employed to test whether frequency of TF-expressing monocytes after LPS stimulation could discriminate patients with previous occurrence of VOE from those who had not, as a way to measure strength of association. AUC, area under the curve. (**E**) Frequencies of TF-expressing monocyte subsets was compared were compared between the indicated groups using the Mann-Whitney *U* test. HU group n = 17 and no HU group n = 20. *p < 0.05, ** p < 0.01, ***p < 0.0001.

Aside from producing pro-inflammatory cytokines upon stimulation, monocytes are also able to promote coagulation. Hence, we evaluated production of TF, a central molecule involved in activation of coagulation cascade, in our *in vitro* system. We found that unstimulated cells from both clinical groups displayed similar frequency of TF-expressing monocytes (Fig. 3A). Upon LPS-driven activation, percentage of TF-expressing monocytes was dramatically increased in patients not undergoing HU treatment but remained unchanged in those using HU (Fig. 3A). We did not find differences in mean fluorescence intensity values between the clinical groups and experimental conditions, which indicates that rather than interfering with magnitude protein production per cell basis, HU affected the expansion of cells expressing TF.

### TF-expressing monocytes are associated to vaso-occlusive events

Additional analyses revealed that activated TF-expressing monocytes were associated with previous occurrence of VOE (Fig. 3C). ROC and C-statistics analyses were used to evaluate the association between VOE and TF-expressing monocytes. The greater the area under the ROC curve (AUC) the better the model is at discriminating between increased TF^+^ monocytes frequency and patients who had VOE from those who had not. Patients who had previous history of VOE had increased frequency of TF-expressing monocytes (Fig. 3D). This finding indicated that frequency of TF-expressing monocytes may serve as a biomarker of VOE. We next, we evaluated the ability of the distinct monocyte subtypes to produce TF. Interestingly, in unstimulated cells, the HU therapy was associated with decreased frequency of TF-expressing CD14^+^CD16^+^ monocytes (Fig. 3E). However, LPS stimulation induced an increased in the frequency of TF-expressing CD14^++^CD16^−^, in patients not undergoing HU treatment compared with that using HU (Fig. 3E). These results uncover differential ability to induce TF expression among the distinct subsets of monocytes in SCA patients.

### Capacity of monocyte to produce multiple inflammatory cytokines is affected by hydroxyurea

**Figure 4:**
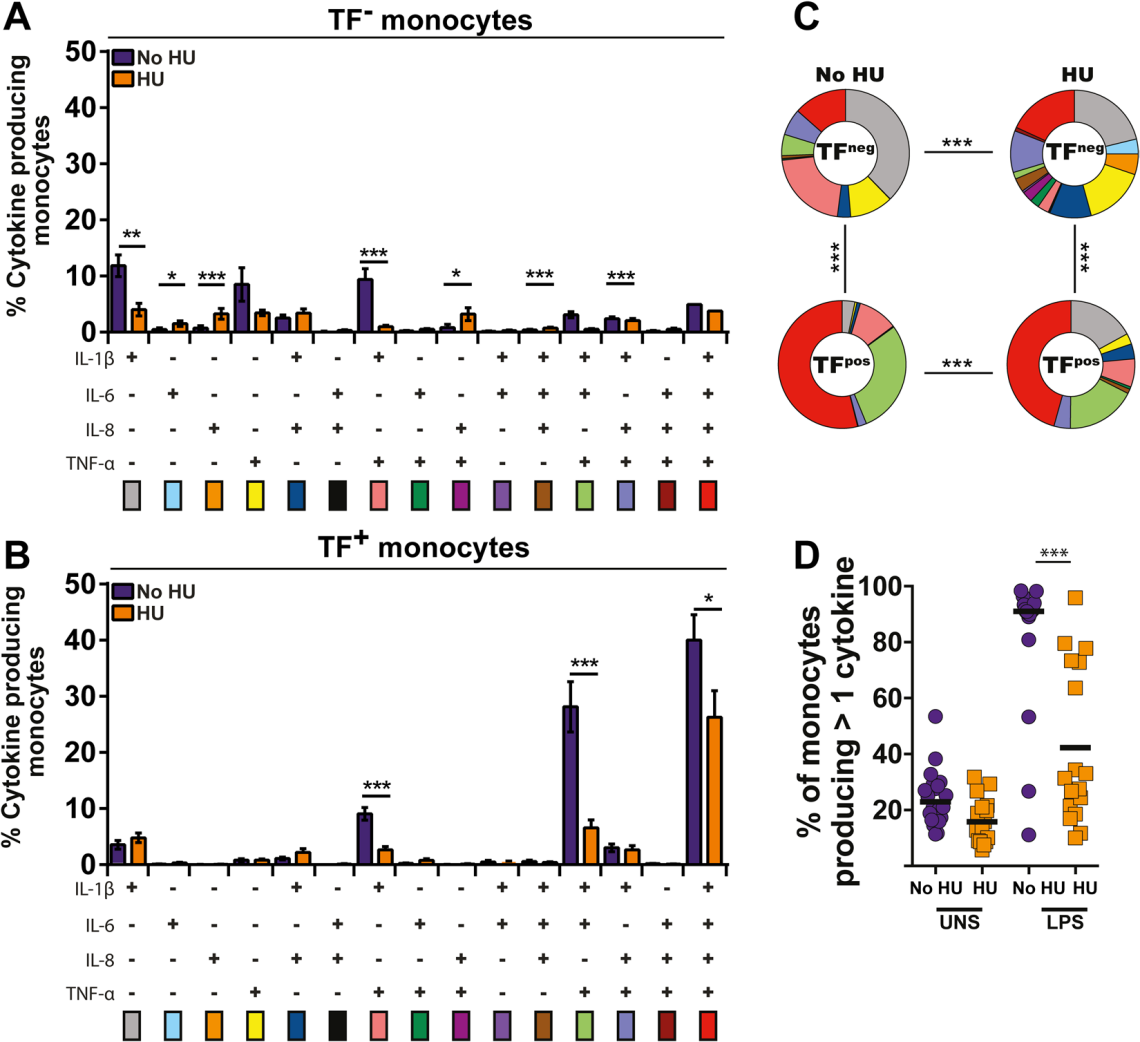
Hydroxyurea therapy reduces the capacity of activated monocytes to produce multiple pro-inflammatory cytokines. Polyfunctional analysis of TF^−^ (**A**) and TF^+^ (**B**) monocytes upon LPS stimulation was performed in PBMC from SCA patients undertaking or not hydroxyurea. Data were compared using the Mann-Whitney *U* test. *p < 0.05, **p < 0.01, ***p < 0.0001. (**C**) The overall cytokine expression profiles of activated TF^−^ or TF^+^ monocytes from SCA patients treated or not with hydroxyurea were compared using the chi-square test. ***p < 0.0001. (**D**) Frequencies of monocytes producing more than 1 cytokine *in vitro* were compared between SCA patients undertaking (n = 17) or not (n = 20) hydroxyurea using the Mann-Whitney *U* test. ***p < 0.0001.

We next examined the capacity of monocytes to produce multiple pro-inflammatory cytokines simultaneously upon LPS-driven activation *in vitro*. Upon stimulation, TF^−^ monocytes from patients who were not taking HU predominantly produced IL-1β, TNF-α or both cytokines simultaneously (Fig. 4A). On the other hand, in the same clinical group, TF^+^ monocytes exhibited the ability to produce more frequently IL-1β, IL-6, IL-8 and TNF-α simultaneously. Interestingly, HU therapy reduced the capacity of monocytes to produce multiple cytokines upon activation (Fig. 4A,B). Thus, the overall function profile in terms of cytokine production was different between TF^−^ and TF^+^ and also between the two clinical groups stratified by HU therapy (Fig. 4C). The frequency of monocytes producing more than one cytokine after the LPS challenge was statistically different, and this polyfunctionality was shown to be dramatically reduced in the monocytes of patients who were taking HU (Fig. 4D).

### Frequency of classical monocytes *ex vivo* and capacity to produce pro-inflammatory cytokines upon LPS stimulation *in vitro*

**Figure 5:**
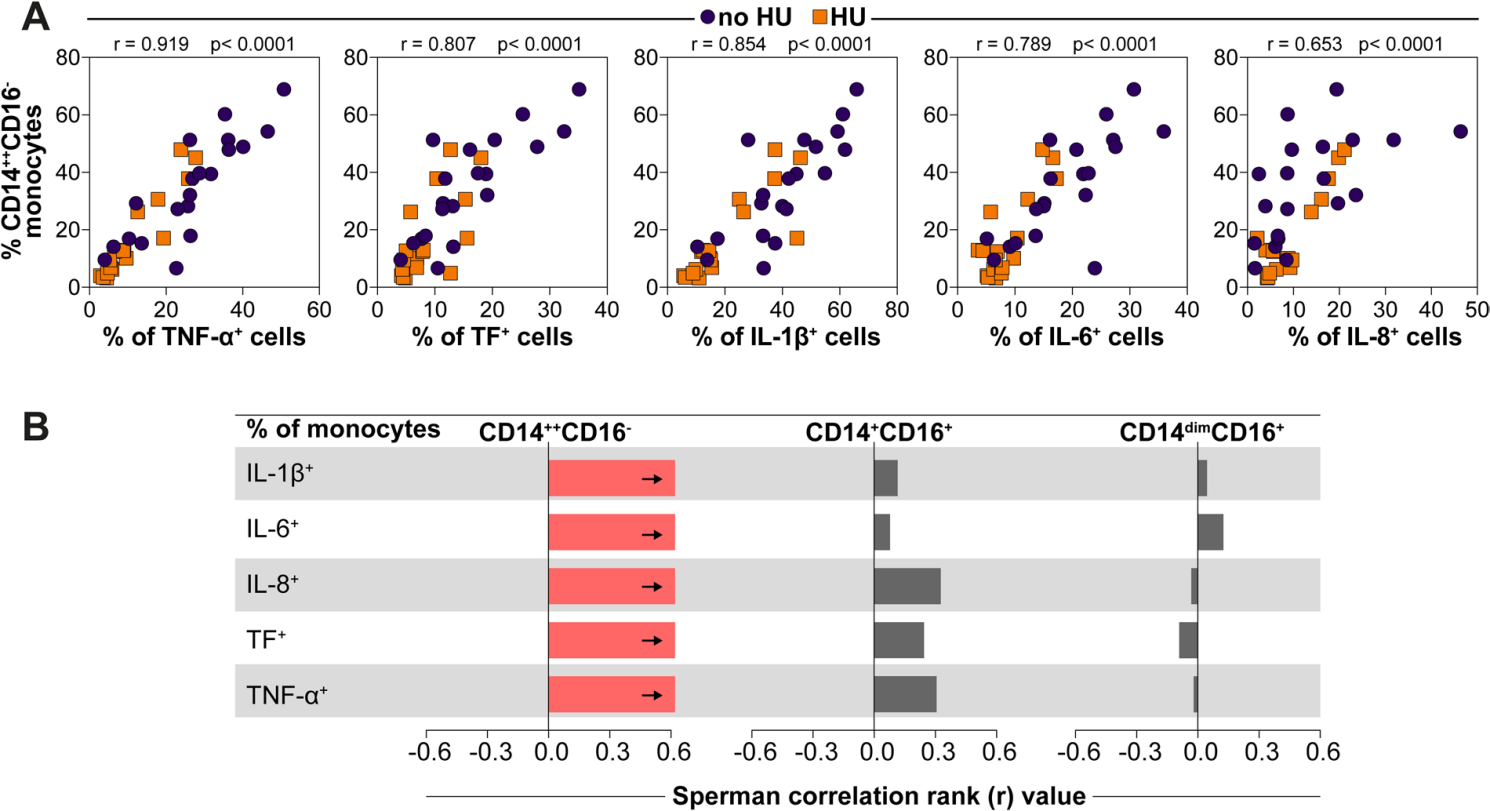
*Ex vivo* frequency of CD14^++^CD16^−^ monocytes in peripheral blood directly correlates with the capacity of activated monocytes to produce pro-inflammatory cytokines. (**A**) Frequencies of classical monocytes in peripheral blood from SCA patients undertaking or not hydroxyurea were tested for correlations with frequencies of monocytes expressing indicated markers after LPS stimulation *in vitro*. Data were compared using the Spearman correlation rank test. (**B**) Spearman correlations between frequency of monocytes subsets and frequency of monocytes expressing IL-1 β, IL-6, IL-8, TF, or TNF-α in SCA patients. HU group n = 17 and no HU group n = 20. Bars represent the strength of correlation (r values). Red bar indicates statistically significant correlation (p < 0.05 after adjustment for multiple comparisons) while grey bars were nonsignificant.

After assessing monocytes polyfunctionality, we sought to see whether frequency of classical monocytes in peripheral blood *ex vivo* was associated with capacity to produce pro-inflammatory cytokines upon LPS stimulation *in vitro*. Spearman correlation analyses revealed that frequency of monocytes expressing CD14^++^CD16^−^ in the entire study population exhibited strong positive association with percentage of monocytes expressing TNF-α^+^, TF^+^, IL-1β^+^, IL-6^+^ and IL-8^+^ upon LPS stimulation (Fig. 5A). Noteworthy, in patients undergoing HU therapy reduction of CD14^++^CD16^−^ frequencies was proportional to reduction of cytokine production (Fig. 5A), implicating the classical monocyte subset was a potential source of such pro-inflammatory molecules. Of note, frequencies of the other monocyte subsets did not significantly correlated with the frequency of cells expressing these inflammatory mediators (Fig. 5B).

## Discussion

Chronic inflammation and persistent activation of coagulation, with systemic involvement, are main features of SCA. This disease has a high prevalence and incidence worldwide and a very complex pathophysiology^1^. Although HU is considered the main therapeutic option for SCA, the specific mechanisms leading to improvement of clinical manifestations is not completely described. As previously described^25–27^, HU therapy has been associated with improvement of hemolysis markers, increased HbF and decreased HbS levels as well as reduction of monocyte counts. Our results are in agreement with a previous study reporting that HU therapy reduced frequency of VOE and pain crisis^27^.

The biological relevance of the role of monocytes in SCA has been previously demonstrated, such as involvement with VOE^14,15^. Nevertheless, details regarding monocytes subsets, activation pattern and cytokine production profile in SCA are not fully understood. Frequency of monocytes subsets identified herein are in agreement with previous characterization in healthy peripheral blood, where the majority has classical phenotype whereas around, 6.7% exhibits intermediate and 9.3% non-classic markers^7^. In contrast, a previous study has found that 75% of monocytes from patients with SCA exhibit a CD14^+^CD16^+^ pro-inflammatory phenotype^28^. Differences in study populations and/or methodological in gating strategy during flow cytometry assays could at least in part explain these discrepancies. Our results demonstrated that HU therapy decreases frequency of classical monocytes (CD14^++^CD16^−^) while increasing percentage of non-classical monocytes (CD14^dim^CD16^+^). Previous studies have shown that HU increases frequency of non-classical monocytes^29^ although the activation status of this subset has not been evaluated. We hypothesize that HU may directly induce differentiation of classical monocytes into non-classical/patrolling phenotype by increasing CD16 expression. Future studies are warranted to answer this question.

The specific pathways driving monocyte activation in SCA are not entirely elucidated. Previous studies have suggested participation of some agonists of toll-like receptor 4 (TLR4), such as free heme^30^, high mobility group box 1 (HMGB1)^31^ and heparan sulfate^32^. It is already known that monocyte activation through TLR4 leads to increased production of TNF-α^33^ which can amplify TF and VCAM-1 expression in endothelial cells^33^. Our experiments demonstrated that upon LPS challenge, monocytes from SCA individuals who were not under HU therapy exhibit increased expression of TNF-α, IL-1β and IL-6 compared to that from those who were taking the drug. Furthermore, unstimulated monocytes from SCA individuals who were not under HU therapy already exhibited increased expression of IL-8. Therefore, the production of pro-inflammatory cytokines seems to be strongly modulated by HU^34^. Several studies include monocyte-activating molecules (such as LPS, TNF-α) in order to increase the responsiveness of the cells and to emphasize an activated phenotype^7,35^. Monocytes were obtained from patients in steady-state (in absence of inflammatory crisis), thus we decided to challenge the cells with LPS, in order to increase cytokine and TF production and to mimic an activation process. We found that monocytes from patients treated with HU produced less cytokine, which allows us to suggest that although LPS is able to activate the cells; their response is damped by the HU therapy. It has been shown that HU decreases levels of TNF-α, IL-8^19^, IL-1β^36^ and IL-6^37^ in both plasma and serum of SCA individuals. More recently, it has been demonstrated that heme is able to increase IL-6 expression in SCA monocytes, since addition of iron chelator decreased its expression^38^. Considering that heme is released during hemolysis, these findings argue that intravascular hemolysis may play a pivotal role in monocyte activation in SCA. Collectively, these data indicate that HU affects not only monocyte subsets but also the ability of these cells to produce pro-inflammatory cytokines.

TF production at sites of vascular damage promotes the activation of VII factor, thrombin generation and fibrin deposition^39^. The mechanism underlying TF production and expression in both endothelial cells and monocytes has been extensively investigated in SCA^40^. A suggested that heme from intravascular hemolysis can activate endothelial cells and leading to NF-kB nuclear translocation^40^. These events promote transcription of adhesion molecules such as P-selectin and pro-inflammatory cytokines^40^. The participation of TF from monocytes and endothelial cells on VOE has also been related to microparticles production during steady state, and it is dramatically augmented during crisis^41^, which can contribute to VOE. Here we found that HU therapy reduced TF expression by activated monocytes in patients undergoing treatment, corroborating with previous findings demonstrating decreased TF protein levels in plasma^42^. Our results further confirmed that TF^+^ monocytes are associated to occurrence of VOE in the study population. TF^+^ monocytes are described to be increased in SCA individuals (HbSS) compared to those with HbSC disease or controls^21^. In addition, frequency of TF^+^ monocytes has been shown to correlate with reticulocyte and leukocyte counts and soluble E-selectin levels^21^. Finally, other studies have shown that percentage of TF^+^ monocytes in peripheral blood increases during VOE^43^.

Immune cells polyfunctionality, in terms of cytokine production, has been recently described in lymphocytes^44^ and monocytes^10^, in the context of infectious diseases. In sterile inflammatory conditions such as SCA, the polyfunctionality still remains to be evaluated. In the present study, we investigated the cytokine profile production of both TF^+^ and TF^−^ monocytes and also tested the effect of HU in production of multiple pro-inflammatory cytokines. Our data provide evidence that patients who were not under HU therapy have increased frequency of monocytes simultaneously producing TF, IL-1β, IL-6, IL-8 and TNF-α. Nonetheless, HU substantially dampened such production without affecting cell death. This result suggests that the inflammatory response promoted by activated monocytes relies on the production of multiple pro-inflammatory cytokines and is directly affected by HU therapy.

Lastly, our correlation analyses revealed that frequency of classical monocytes was positively correlated with percentage of cells producing TF as well as all the inflammatory cytokines examined in the entire study population. The role of classical monocytes on production of pro-inflammatory cytokines has been previously shown in healthy individuals^7^. In hematological diseases such as chronic myelomonocytic leukemia (CMML), it was shown that classical monocytes account for 94% of total monocytes and that this frequency could be useful to distinguish between CMML and reactive monocytosis^45^. A model of lung ischemia-reperfusion injury has shown that classical monocytes were mobilized from the spleen and they also mediated neutrophil extravasation for the sites of injury^46^. During human immunodeficiency virus (HIV) infection, classical monocytes were shown to have increased capacity to promote activation of TF and to produce multiple pro-inflammatory cytokines suggesting their ability to crosstalk coagulation and inflammation^10^. Regarding sickle cell disease, previous evaluation of monocytes subsets has identified that non-classical or patrolling monocytes express low levels of TNF-α and IL-6 and they seem to be important protecting the microvasculature from VOE^35^. To our knowledge, this is the first study to determine *ex vivo* characterization of monocytes subsets and to identify their polyfunctionality in SCA. Of note, the association between TF-expressing monocytes and occurrence of VOE also highlights the importance of these cells in vascular complications linked to SCA.

In summary, our data corroborate with previous studies that show beneficial effects of HU therapy in SCA. We show that HU is associated with the improvement of laboratory parameters, to decreased frequency and activation of the classical inflammatory monocytes. Importantly, HU therapy directly dampened the polyfunctional capacity of monocytes, suggesting an overall anti-inflammatory property which the molecular mechanism still requires elucidation. Considerations regarding monocytes subsets, activation profile and cytokine production are useful to suggest novel therapeutic targets and may help to understand the inflammatory mechanism underlying SCA.

## Material and Methods

### Subjects

Thirty-seven pediatric SCA patients (HbSS genotype) were enrolled in the present study, eighteen (48.6%) of whom were female, all seen at the Bahia Hemotherapy and Hematology Foundation from August 2017 to December 2017. The patients had an average age of 14.16 ± 3.08 years and a median age of 14 years (interquartile range [IQR]: 12–17 years). All patients were in steady state of sickle cell anemia, characterized as the absence of acute crisis in the past three months prior to blood collection procedures. Three patients have had stroke and were under blood transfusion therapy; one patient had received transfusion 10 months before study enrollment and the other two had received 30 days before enrollment. Twenty patients were not under HU therapy while 17 patients were taking HU for at least 5 months. Prior to enrollment in the study informed consent was obtained from all individual participants. Legal guardians agreed to allow the biological sample collection procedures and signed terms of informed consent of all individuals under 18 years, while individuals older than 18 years have signed the assent form. This study received approval from the Institutional Research Board of São Rafael Hospital (protocol number: 1400535) and is in compliance with the ethical principles of the revised Declaration of Helsinki.

### Clinical manifestations

At the time of enrollment, clinical data regarding the occurrence of previous clinical manifestations (e.g. VOE) were collected using a standardized questionnaire (self-reported or reported by the parents) and confirmed by the medical records. Patients or their legal guardians were asked whether they ever had or not, during their lifetime, any clinical manifestation related to SCA. Hospital admissions were defined as hospitalization for more than three days and VOE were defined as acute pain affecting any body part lasting several hours in association with swelling especially in the joints and soft tissues requiring medication. Patients with previous history of VOE presented at least one episode of VOE (ranging from 1 to 5 events) in the past six months.

### Laboratory characterization

Hematological parameters were obtained using a Beckman Coulter LH 780 Hematology Analyzer (Beckman Coulter, Brea, California, USA) and hemoglobin patterns were confirmed by high-performance liquid chromatography employing an HPLC/Variant-II hemoglobin testing system (Bio-Rad, Hercules, California, USA). Biochemical parameters, including lipid profile, total bilirubin and fractions, lactate dehydrogenase, iron, hepatic metabolism and renal profile were determined using an automated A25 chemistry analyzer (Biosystems S.A, Barcelona, Catalunya, Spain). Ferritin levels were determined using Access 2 Immunochemistry System (Beckman Coulter Inc., Pasadena, California, USA). C-reactive protein and alpha-1 antitrypsin levels were measured using IMMAGE® Immunochemistry System (Beckman Coulter Inc., Pasadena, California, USA). Laboratory parameters were analyzed at the Clinical Analyses Laboratory of the College of Pharmaceutical Sciences (Universidade Federal da Bahia).

### *Ex vivo* monocyte phenotyping by flow cytometry

Fresh peripheral blood mononuclear cells (PBMC) were obtained from SCA patients’ blood samples collected with heparin, through gradient centrifugation on Ficoll Paque Plus (Gibco, GE Healthcare Bio-Sciences Corp. Piscataway, NJ, USA) at room temperature. Isolated PBMC was cryopreserved in 90% of fetal bovine serum (FBS, Gibco, GE Healthcare Bio-Sciences Corp. Piscataway, NJ, USA) and 10% of DMSO (Sigma, St. Louis, MO, USA) until flow cytometry assay. All the samples were processed within one hour after collection. PBMC were thawed and resuspended in RPMI 1640 supplemented with 10% FBS at 10^6^ cells per well in 96-well plates. Cells were washed and resuspended in complete media with Brefeldin-A (Biolegend, San Diego, California, USA) and Monensin (Biolegend, San Diego, California, USA), two molecules capable to stop Golgi apparatus and vesicles secretion^47,48^, in order to block cytokine secretion and stimulated with 100 ng/mL of LPS, a well-known TLR4 agonist in order to increase cytokine and TF expression (Sigma, St. Louis, MO, USA) for 6 hours at 37 °C in 5% CO2. Following stimulation, extracellular staining of phenotypic markers was performed. Monocyte immunophenotyping was carried out by detection of CD14 (Qdot 605), CD16 (PE-Cy7), HLA-DR (APC-Cy7) on cell surface. Several lineage markers including CD2, CD3, CD19, CD20, CD56 (Pacific Blue) were used to exclude other cells aside from monocytes of the analyses (see flow cytometry example plots in Supplementary Fig. 1). Dead cells and debris were also excluded by using Aqua fluorescent reactive Live/Dead dye (ThermoFisher Scientific, Waltham, MA, USA). Based on CD14 and CD16 surface expression, three monocyte subsets were examined: classical/inflammatory (CD14^++^CD16^−^), intermediate (CD14^+^CD16^+^) and non-classical (CD14^dim^CD16^+^) monocytes. To determine monocyte functionality, cells were fixed and permeabilized using the Intracellular Fixation & Permeabilization Buffer Set from eBioscience (ThermoFisher) and intracellular staining was performed detecting TNF-α (PerCP-Cy5.5), TF (APC), IL-8 (FITC), IL-1β (PE) and IL-6 (AF-700). Our results of flow cytometry assay are described as percentage of positive cells among HLADR^+^DUMP^−^ cells (which in the present investigation are denominated “monocytes”, as described in overall gating strategy in the Fig. S1), per a total of 10^6^ PBMC/well for each experiment. Description of antibody clones, conjugated fluorochromes, catalog numbers and dilutions used is shown in Table S3. Antibodies dilutions were carried out according to each manufacturer’s instructions and validated in titration experiments. Acquisition of the stained cells was performed using a BD LSRFortessa™ cell analyzer (BD Bioscience, San Jose, CA, USA) and Software FlowJo, LLC (BD Bioscience, San Jose, CA, USA) was used to analyze the data.

### Statistical analysis

Statistical analyses were performed using the Statistical Package for the Social Sciences (SPSS) version 20.0 software (IBM, Armonk, New York, USA), JMP software v.12 (SAS Institute, Cary, North Carolina, USA) and GraphPad Prism version 6.0 (Graphpad Software, San Diego, California, USA), which was also used to assemble the graphs. Baseline values of selected variables are expressed as means with their respective standard variation. The Shapiro-Wilk test was used to determine variable distribution. The Mann-Whitney *U* test and independent t-test were used to compare the groups according to the normality of the distribution for each variable. Fisher’s exact test was used to compare frequency of clinical manifestations as well as sex distribution between the patients groups. Spearman correlation rank analysis was performed to test correlations between frequency of monocyte subsets and cytokine production profiles. Results were adjusted for multiple comparisons using Bonferroni’s method. Receiver Operator Characteristics (ROC) curve analysis was used to test the association between frequency of TF-expressing monocytes in blood and occurrence of VOE. Pearson’s qui-square test was employed to compare the polyfunctionality profiles of monocytes^10^. All analyses were pre-specified. P values < 0.05, after correction for multiple measurements using the Holm-Bonferroni method were considered statistically significant.

## Supplementary information

Supplementary Material

## References

[1] Kato GJ (2018). Sickle cell disease. Nature reviews. Disease primers.

[2] Guarda CCD (2017). Heme-mediated cell activation: the inflammatory puzzle of sickle cell anemia. Expert review of hematology.

[3] Manwani D, Frenette PS (2013). Vaso-occlusion in sickle cell disease: pathophysiology and novel targeted therapies. Blood.

[4] Henson PM, Hume DA (2006). Apoptotic cell removal in development and tissue homeostasis. Trends in immunology.

[5] Kratofil RM, Kubes P, Deniset JF (2017). Monocyte Conversion During Inflammation and Injury. Arteriosclerosis, thrombosis, and vascular biology.

[6] Ingersoll MA, Platt AM, Potteaux S, Randolph GJ (2011). Monocyte trafficking in acute and chronic inflammation. Trends in immunology.

[7] Boyette LB (2017). Phenotype, function, and differentiation potential of human monocyte subsets. PloS one.

[8] Grage-Griebenow E, Flad HD, Ernst M (2001). Heterogeneity of human peripheral blood monocyte subsets. Journal of leukocyte biology.

[9] Grun JL (2018). High-Density Lipoprotein Reduction Differentially Modulates to Classical and Nonclassical Monocyte Subpopulations in Metabolic Syndrome Patients and in LPS-Stimulated Primary Human Monocytes *In Vitro*. Journal of immunology research.

[10] Schechter Melissa E., Andrade Bruno B., He Tianyu, Richter George Haret, Tosh Kevin W., Policicchio Benjamin B., Singh Amrit, Raehtz Kevin D., Sheikh Virginia, Ma Dongying, Brocca-Cofano Egidio, Apetrei Cristian, Tracy Russel, Ribeiro Ruy M., Sher Alan, Francischetti Ivo M. B., Pandrea Ivona, Sereti Irini (2017). Inflammatory monocytes expressing tissue factor drive SIV and HIV coagulopathy. Science Translational Medicine.

[11] Andrade BB (2014). Mycobacterial antigen driven activation of CD14++CD16- monocytes is a predictor of tuberculosis-associated immune reconstitution inflammatory syndrome. PLoS pathogens.

[12] Woollard KJ, Geissmann F (2010). Monocytes in atherosclerosis: subsets and functions. Nature reviews. Cardiology.

[13] Nahrendorf M, Pittet MJ, Swirski FK (2010). Monocytes: protagonists of infarct inflammation and repair after myocardial infarction. Circulation.

[14] Belcher JD, Marker PH, Weber JP, Hebbel RP, Vercellotti GM (2000). Activated monocytes in sickle cell disease: potential role in the activation of vascular endothelium and vaso-occlusion. Blood.

[15] Safaya S, Steinberg MH, Klings ES (2012). Monocytes from sickle cell disease patients induce differential pulmonary endothelial gene expression via activation of NF-kappaB signaling pathway. Molecular immunology.

[16] Carvalho Magda O. S., Araujo-Santos Théo, Reis João H. O., Rocha Larissa C., Cerqueira Bruno A. V., Luz Nívea F., Lyra Isa M., Lopes Valma M., Barbosa Cynara G., Fiuza Luciana M., Santiago Rayra P., Figueiredo Camylla V. B., da Guarda Caroline C., Barral Neto Manoel, Borges Valéria M., Gonçalves Marilda S. (2017). Inflammatory mediators in sickle cell anaemia highlight the difference between steady state and crisis in paediatric patients. British Journal of Haematology.

[17] Brittain JE, Parise LV (2007). Cytokines and plasma factors in sickle cell disease. Current opinion in hematology.

[18] Cajado C (2011). TNF-alpha and IL-8: serum levels and gene polymorphisms (−308G>A and −251A>T) are associated with classical biomarkers and medical history in children with sickle cell anemia. Cytokine.

[19] Lanaro C (2009). Altered levels of cytokines and inflammatory mediators in plasma and leukocytes of sickle cell anemia patients and effects of hydroxyurea therapy. Journal of leukocyte biology.

[20] Spronk HM, ten Cate H, van der Meijden PE (2014). Differential roles of tissue factor and phosphatidylserine in activation of coagulation. Thrombosis research.

[21] Setty BN (2012). Tissue factor-positive monocytes in children with sickle cell disease: correlation with biomarkers of haemolysis. British journal of haematology.

[22] Solovey A, Gui L, Key NS, Hebbel RP (1998). Tissue factor expression by endothelial cells in sickle cell anemia. The Journal of clinical investigation.

[23] Steinberg MH (2003). Effect of hydroxyurea on mortality and morbidity in adult sickle cell anemia: risks and benefits up to 9 years of treatment. Jama.

[24] Charache S (1996). Hydroxyurea and sickle cell anemia. Clinical utility of a myelosuppressive “switching” agent. The Multicenter Study of Hydroxyurea in Sickle Cell Anemia. Medicine.

[25] Rodgers GP, Dover GJ, Noguchi CT, Schechter AN, Nienhuis AW (1990). Hematologic responses of patients with sickle cell disease to treatment with hydroxyurea. The New England journal of medicine.

[26] Yahouédéhou SCMA (2018). Sickle Cell Anemia Patients in Use of Hydroxyurea: Association between Polymorphisms in Genes Encoding Metabolizing Drug Enzymes and Laboratory Parameters. Disease Markers.

[27] Wang WC (2011). Hydroxycarbamide in very young children with sickle-cell anaemia: a multicentre, randomised, controlled trial (BABY HUG). The Lancet.

[28] Singhal R (2017). Development of pro-inflammatory phenotype in monocytes after engulfing Hb-activated platelets in hemolytic disorders. Clin Immunol.

[29] Yotsumoto Fertrin K (2012). Monocyte Shift to a Non-Classical CD14dim/CD16+ Phenotype Correlates with Fetal Hemoglobin Levels in Sickle Cell Anemia Patients Treated with Hydroxyurea. Blood.

[30] Figueiredo RT (2007). Characterization of heme as activator of Toll-like receptor 4. The Journal of biological chemistry.

[31] Kim S (2013). Signaling of high mobility group box 1 (HMGB1) through toll-like receptor 4 in macrophages requires CD14. Mol Med.

[32] Akbarshahi H (2011). TLR4 dependent heparan sulphate-induced pancreatic inflammatory response is IRF3-mediated. Journal of translational medicine.

[33] Solovey A (2017). A monocyte-TNF-endothelial activation axis in sickle transgenic mice: Therapeutic benefit from TNF blockade. American journal of hematology.

[34] Penkert RR (2018). Inflammatory molecule reduction with hydroxyurea therapy in children with sickle cell anemia. Haematologica.

[35] Liu Y (2018). HO-1(hi) patrolling monocytes protect against vaso-occlusion in sickle cell disease. Blood.

[36] Keikhaei B (2013). Altered levels of pro-inflammatory cytokines in sickle cell disease patients during vaso-occlusive crises and the steady state condition. European cytokine network.

[37] Bandeira IC (2014). Chronic inflammatory state in sickle cell anemia patients is associated with HBB(*)S haplotype. Cytokine.

[38] Dagur PK (2018). Haem augments and iron chelation decreases toll-like receptor 4 mediated inflammation in monocytes from sickle cell patients. British journal of haematology.

[39] Ito T (2014). PAMPs and DAMPs as triggers for DIC. Journal of intensive care.

[40] Belcher JD (2014). Heme triggers TLR4 signaling leading to endothelial cell activation and vaso-occlusion in murine sickle cell disease. Blood.

[41] Shet AS (2003). Sickle blood contains tissue factor-positive microparticles derived from endothelial cells and monocytes. Blood.

[42] Colella MP (2012). Hydroxyurea is associated with reductions in hypercoagulability markers in sickle cell anemia. Journal of thrombosis and haemostasis: JTH.

[43] Soliman M, Ragab S (2015). Tissue factor-positive monocytes in children with sickle cell disease: relation to vaso-occlusive crisis. The Egyptian Journal of Haematology.

[44] Hsu Denise C, Breglio Kimberly F, Pei Luxin, Wong Chun-Shu, Andrade Bruno B, Sheikh Virginia, Smelkinson Margery, Petrovas Constantinos, Rupert Adam, Gil-Santana Leonardo, Zelazny Adrian, Holland Steven M, Olivier Kenneth, Barber Daniel, Sereti Irini (2018). Emergence of Polyfunctional Cytotoxic CD4+ T Cells in Mycobacterium avium Immune Reconstitution Inflammatory Syndrome in Human Immunodeficiency Virus-Infected Patients. Clinical Infectious Diseases.

[45] Selimoglu-Buet D (2017). Accumulation of classical monocytes defines a subgroup of MDS that frequently evolves into CMML. Blood.

[46] Hsiao Hsi-Min, Fernandez Ramiro, Tanaka Satona, Li Wenjun, Spahn Jessica H., Chiu Stephen, Akbarpour Mahzad, Ruiz-Perez Daniel, Wu Qiang, Turam Cem, Scozzi Davide, Takahashi Tsuyoshi, Luehmann Hannah P., Puri Varun, Budinger G.R. Scott, Krupnick Alexander S., Misharin Alexander V., Lavine Kory J., Liu Yongjian, Gelman Andrew E., Bharat Ankit, Kreisel Daniel (2018). Spleen-derived classical monocytes mediate lung ischemia-reperfusion injury through IL-1β. Journal of Clinical Investigation.

[47] Chardin P, McCormick F, Brefeldin A (1999). the advantage of being uncompetitive. Cell.

[48] Mollenhauer HH, Morre DJ, Rowe LD (1990). Alteration of intracellular traffic by monensin; mechanism, specificity and relationship to toxicity. Biochimica et biophysica acta.

