# Supplementary material for: Hydroxyurea alters circulating monocyte subsets and dampens its inflammatory potential in sickle cell anemia patients
Source: Sci Rep. 2019 Oct 15;9:14829. doi: 10.1038/s41598-019-51339-x (PMC6794261; doi:10.1038/s41598-019-51339-x)
Supplement: Supplementary file 1 — Supplementary Material [file 41598_2019_51339_MOESM1_ESM.docx]

**Hydroxyurea alters circulating monocyte subsets and dampens its inflammatory potential in sickle cell anemia patients**

Caroline C. Guarda^1,2*^, Paulo S. M. Silveira-Mattos^2,3,4*^, Sètondji C. M. A. Yahouédéhou^1,2^, Rayra P. Santiago^1,2^, Milena M. Aleluia^1^, Camylla V. B. Figueiredo^1,2^, Luciana M. Fiuza^1,2^, Suellen P. Carvalho^1,2^, Rodrigo M. Oliveira^1,2^, Valma M. L. Nascimento^5^, Nívea F. Luz^3^, Valéria M. Borges^2,3^, Bruno B. Andrade^2,3,4*^ and Marilda S. Gonçalves^1,2*†^

| **Table S1.** Association of laboratory parameters in SCA patients taking or not HU | | | |
| --- | --- | --- | --- |
| **Characteristics** | **No HU (N = 20)** | **HU (N = 17)** | ***P* value** |
|  | Mean ± SD | Mean ± SD |  |
| Sex |  |  |  |
| Females | 11 (55.0%) | 7 (41.2%) | - |
| Male | 9 (45.0%) | 10 (58.8%) |  |
| Age, years | 15 ± 3 | 13 ± 2 | - |
|  |  |  |  |
| **Hemolysis markers** |  |  |  |
| RBC, 10^6^/mL | 2.62 ± 0.29 | 2.56 ± 0.38 | 0.612 |
| Hemoglobin, g/dL | 8.15 ± 0.72 | 8.80 ± 1.08 | **0.045**^#^ |
| Hematocrit, % | 23.85 ± 2.21 | 26.24 ± 3.34 | **0.013** |
| MCV, fL | 88.54 ± 7.88 | 101.19 ± 8.11 | **0.000** |
| MCH, ρg | 30.55 ± 2.61 | 34.57 ± 3.64 | **0.000** |
| MCHC, g/dL | 34.51 ± 0.83 | 33.54 ± 0.74 | **0.001** |
| RDW, % | 24.02 ± 0.97 | 19.71 ± 0.53 | **0.001** |
| Reticulocyte count, % | 6.93 ± 2.50 | 5.39 ± 2.83 | 0.089 |
| Total bilirubin, mg/dL | 3.25 ± 1.09 | 2.72 ± 1.51 | 0.231 |
| Direct bilirubin, mg/dL | 0.44 ± 0.19 | 0.43 ± 0.14 | 0.895 |
| Indirect bilirubin, mg/dL | 2.81 ± 1.09 | 2.29 ± 1.50 | 0.236 |
| LDH, U/L | 1304.05 ± 361.27 | 984.59 ± 296.83 | **0.007** |
|  |  |  |  |
| **Hb pattern** |  |  |  |
| HbS, % | 87.42 ± 9.26 | 82.85 ± 5.27 | **0.014^#^** |
| HbF, % | 6.78 ± 5.13 | 12.86 ± 5.86 | **0.003** |
|  |  |  |  |
| **Leukocytes** |  |  |  |
| WBC /mL | 12499.00 ± 2798.02 | 11371.76 ± 3716.55 | 0.300 |
| Neutrophils /mL | 6180.90 ± 2590.30 | 5849.18 ± 2916.16 | 0.537^#^ |
| Monocytes /mL | 1360.55 ± 622.39 | 966.29 ± 516.90 | **0.046** |
| Eosinophils /mL | 490.42 ± 245.36 | 343.65 ± 230.94 | 0.074 |
| Basophil /mL | 91.65 ± 77.35 | 92,06 ± 118.36 | 0.497^#^ |
| Lymphocytes /mL | 4087.25 ± 1133.09 | 3939.82 ± 988.26 | 0.679 |
|  |  |  |  |
| **Platelets** |  |  |  |
| Platelet count, x10^3^/mL | 440.25 ± 69.04 | 413.00 ± 134.63 | 0.458 |
| MPV, fL | 8.16 ± 0.84 | 7.94 ± 1.00 | 0.490 |
| PCT, % | 0.35 ± 0.06 | 0.30 ± 0.08 | 0.067 |
| PDW, % | 16.24 ± 0.37 | 15.98 ± 0.17 | **0.026** |
|  |  |  |  |
| **Lipid metabolism** |  |  |  |
| Total Cholesterol, mg/dL | 125.37 ± 25.83 | 115.29 ± 23.67 | 0.233 |
| HDL-C, mg/dL | 34.95 ± 8.67 | 38.12 ± 8.14 | 0.268 |
| LDL-C, mg/dL | 70.35 ± 19.67 | 59.67 ± 19.20 | 0.081^#^ |
| VLDL-C, mg/dL | 20.06 ± 8.57 | 17.50 ± 5.22 | 0.283 |
| Triglycerides, mg/dL | 100.32 ± 42.89 | 87.53 ± 26.13 | 0.283 |
|  |  |  |  |
| **Iron metabolism** |  |  |  |
| Iron, mcg/dL | 95.32 ± 31.24 | 132.18 ± 60.47 | **0.038**^#^ |
| Ferritin, *η*g/mL | 259.7 ± 490.8 | 673.4 ± 468.9 | **0.001^#^** |
|  |  |  |  |
| **Renal profile** |  |  |  |
| Urea, mg/dL | 16.63 ± 4.94 | 20.21 ± 7.56 | 0.285^#^ |
| Creatinine, mg/dL | 0.41 ± 0.16 | 0.47 ± 0.11 | 0.212 |
| Uric Acid, mg/dL | 3.96 ± 1.17 | 3.66 ± 1.21 | 0.464 |
|  |  |  |  |
| **Hepatic profile** |  |  |  |
| AST, U/L | 52.95 ± 17.22 | 37.53 ± 11.95 | **0.004** |
| ALT, U/L | 21.26 ± 12.06 | 15.59 ± 7.04 | 0.271^#^ |
| GGT, U/L | 21.53 ± 10.32 | 19.88 ± 9.08 | 0.683^#^ |
| Alkaline phosphatase, U/L | 145.00 ± 88.64 | 120.76 ± 43.26 | 0.573^#^ |
|  |  |  |  |
| **Inflammatory profile** |  |  |  |
| CRP, mg/L | 4.95 ± 5.65 | 4.65 ± 3.55 | 0.845^#^ |
| AAT, mg/dL | 86.77 ± 42.79 | 68.43 ± 45.30 | 0.267^#^ |
|  |  |  |  |

# RBC: red blood cells; MCV: mean cell volume; MCH: mean corpuscular hemoglobin; MCHC: mean corpuscular hemoglobin concentration; RDW: red cell distribution width; LDH: lactate dehydrogenase; HbS: hemoglobin S; HbF: fetal hemoglobin; WBC: white blood cell; MPV: mean platelet volume; PCT: plateletcrit; PDW: platelet distribution width; HDL-C: high-density lipoprotein cholesterol; LDL-C: low-density lipoprotein cholesterol; VLDL-C: very low-density lipoprotein cholesterol; AST: aspartate amino-transferase; ALT: alanine amino-transferase; GGT: gamma glutamyl-transferase; CRP: C-reactive protein; AAT: Alpha-1 antitrypsin. Bold values indicate significance at p<0.05; p-value obtained using t-test. ^#^p-value obtained using Mann-Whitney *U* test.

| **Table S2.** Frequency of clinical manifestations among SCA patients taking or not HU | | | |
| --- | --- | --- | --- |
| **Clinical manifestation** | **No HU**  **(N = 20)** | **HU**  **(N = 17)** | ***P* value** |
| Previous hospital admissions | 16 (80%) | 17 (100%) | 0.109 |
| Pneumonia | 12 (60%) | 9 (52.9%) | 0.746 |
| Splenomegaly | 7 (35%) | 9 (52.9%) | 0.331 |
| Stroke | 2 (10%) | 1 (5.8%) | 1.00 |
| Vaso-occlusive events | 9 (45%) | 2 (11.7%) | **0.036** |
| Infections | 15 (75%) | 14 (82.3%) | 0.701 |
| Priapism | 2 (10%) | 0 (0%) | 0.471 |
| Leg ulcer | 1 (5%) | 1 (5.8%) | 1.00 |
| Acute chest syndrome | 5 (25%) | 4 (23.5%) | 1.00 |
| Cholelithiasis | 5 (25%) | 3 (17.6%) | 0.701 |
| Blood transfusion | 3 (15%) | 2 (11.7%) | 1.00 |

Data were compared using the Fisher’s exact test. Significant p values are shown in bold type font.

# Table S3. List of antibodies used in the flow cytometry experiments.

| **Marker** | **Clone** | **Company** | **Catalog** | **Dilution** |
| --- | --- | --- | --- | --- |
| CD2 | RPA-2.10 | eBioscience | 48-0029-42 | 1:200 |
| CD3 | UCHT1 | eBioscience | 48-0038-42 | 1:25 |
| CD14 | M5E2 | Biolegend | 301834 | 1:100 |
| CD16 | 3G8 | Biolegend | 302015 | 1:100 |
| CD19 | HIB19 | eBioscience | 48-0199-42 | 1:200 |
| CD20 | 2H7 | eBioscience | 48-0209-42 | 1:20 |
| CD56 | B159 | BD Biosciences | 560360 | 1:20 |
| CD142 (TF) | HTF-1 | eBioscience | 17-1429-42 | 1:200 |
| HLA-DR | L243 | BD Biosciences | 641393 | 1:200 |
| IL-1β | JK1B-1 | Biolegend | 508206 | 1:25 |
| IL-6 | MQ2-13A5 | eBioscience | 56-7069-42 | 1:25 |
| TNF-α | MAb11 | Biolegend | 502924 | 1:200 |
| IL-8 | AS14 | BD Biosciences | 340509 | 1:25 |


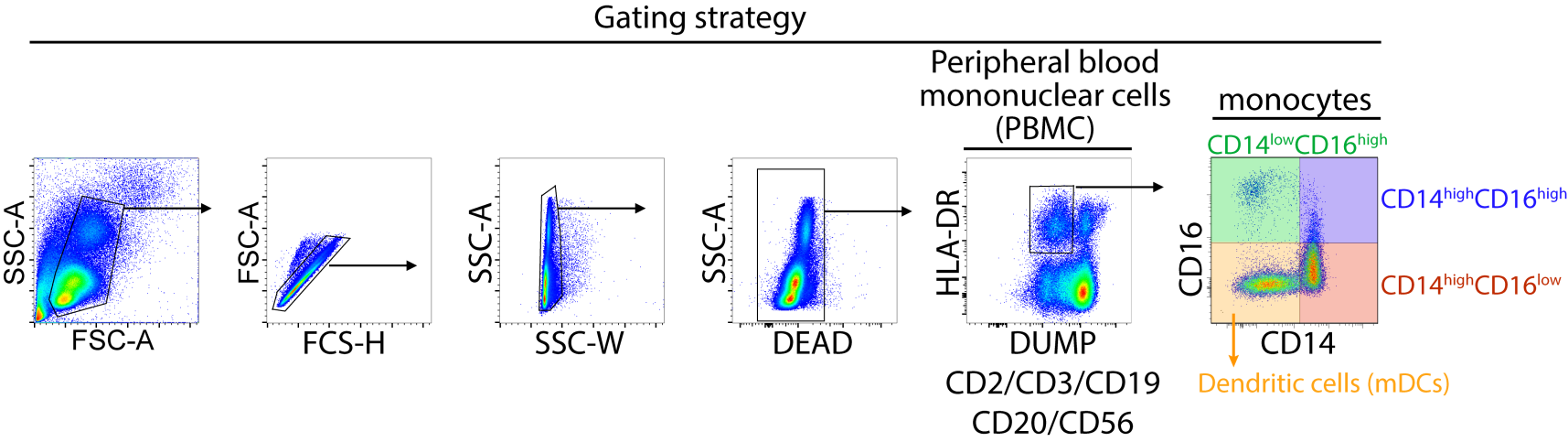


**Figure S1. Overall gating strategy used for flow cytometry assays to evaluate monocytes in peripheral blood.**
